# PP100 A Study On Health-Related Quality Of Life Among Patients With Advanced Gastric Or Gastro-esophageal Junction Adenocarcinoma

**DOI:** 10.1017/S0266462324002629

**Published:** 2025-01-07

**Authors:** Doyeon Jin, Jeonghoon Ahn, Hyorim Lee, Hyerin Seo

## Abstract

**Introduction:**

Health-related quality of Life (HRQoL) is reported as a strong predictor of survival. However, little is known about the relationship between HRQoL and influencing factors of gastric cancer among Korean patients. This study aims to investigate the HRQoL of Korean patients with advanced gastric cancer and identify factors influencing their HRQoL.

**Methods:**

A total of 156 patients with advanced gastric cancer were 1:1 matched for demographics and clinical characteristics between a ramucirumab/paclitaxel combination group and a chemotherapy group. The objective response of the subjects was divided into four categories: partial response (PR), stable disease (SD), progressive disease (PD), and no complete response/no progressive disease (non-CR/non-PD). The six-month changes in HRQoL measured by EuroQol 5-dimension 5-level questionnaire (EQ-5D-5L) were estimated and further investigated for associations with demographic and clinical factors.

**Results:**

The average changes in HRQoL were highest in PR (0.086±0.057), followed by non-CR/non-PD (0.078±0.076), SD (0.035±0.097), and PD (no change). The HRQoL by age group was 0.176±0.088 in subjects 40 years or younger, 0.035±0.071 in the 50s, 0.067±0.072 in the 60s, and −0.003±0.029 in those who were 70 years or older. The HRQoL of females was 0.145±0.136, and for males it was 0.047±0.066. The HRQoL of the non-diabetic group was 0.072±0.095, and for the diabetic group was 0.044±0.057. The HRQoL of the non-hypertensive group was 0.097±0.097, and for the hypertension group was 0.020±0.048.

**Conclusions:**

For advanced gastric cancer patients, the increase of HRQoL was diminished as cancer progresses, age increases, for males, and when comorbidity of diabetes or hypertension exists. The reported HRQoL data is valuable for health technology assessment of advanced gastric cancer agents such as ramucirumab, as this study is a publicly funded neutral study.

